# Thermophilic Proteins as Versatile Scaffolds for Protein Engineering

**DOI:** 10.3390/microorganisms6040097

**Published:** 2018-09-25

**Authors:** Anthony J. Finch, Jin Ryoun Kim

**Affiliations:** Department of Chemical and Biomolecular Engineering, New York University, 6 MetroTech Center, Brooklyn, NY 11201, USA

**Keywords:** thermophilic proteins, protein engineering, protein stability, evolvability

## Abstract

Literature from the past two decades has outlined the existence of a trade-off between protein stability and function. This trade-off creates a unique challenge for protein engineers who seek to introduce new functionality to proteins. These engineers must carefully balance the mutation-mediated creation and/or optimization of function with the destabilizing effect of those mutations. Subsequent research has shown that protein stability is positively correlated with “evolvability” or the ability to support mutations which bestow new functionality on the protein. Since the ultimate goal of protein engineering is to create and/or optimize a protein’s function, highly stable proteins are preferred as potential scaffolds for protein engineering. This review focuses on the application potential for thermophilic proteins as scaffolds for protein engineering. The relatively high inherent thermostability of these proteins grants them a great deal of mutational robustness, making them promising scaffolds for various protein engineering applications. Comparative studies on the evolvability of thermophilic and mesophilic proteins have strongly supported the argument that thermophilic proteins are more evolvable than mesophilic proteins. These findings indicate that thermophilic proteins may represent the scaffold of choice for protein engineering in the future.

## 1. Introduction

The field of protein engineering focuses on rational modification or combinatorial design of proteins to enhance or otherwise alter protein functionality [1]. Each protein has a specific function which is dictated by its amino acid sequence and three-dimensional (or “folded”) structure. Protein engineers intentionally modify these elements by introducing mutations which may alter the structure and functionality of the protein, improve existing functionality or introduce entirely new functionality [1]. This fact motivates the primary challenge facing protein engineers today: an apparent trade-off between protein function and stability [2]. Decades of research has shown that there is a negative correlation between protein stability and function; since one of the primary purposes of protein engineering is to create or optimize protein activity, protein engineers face the challenge of maintaining protein stability at the cost of desired functionality.

Over the course of the past decade and a half, a great deal of research has been done attempting to better understand this negative correlation between protein stability and functionality. The most prevalent explanation for this stems from the fact that the amino acid residues which promote functionality are often destabilizing, either inherently or as a function of their location in the folded protein [2]. It is known that functionality of proteins—enzymes in particular—largely depends upon the presence of reactive polar and/or hydrophilic residues in the active site [2]. Since the active site is often buried within the three-dimensional structure of the protein, it follows that functionality depends upon the inclusion of highly reactive residues in the interior of the protein [2,3]. In fact, protein functionality has been shown to depend directly on the flexibility of the active site rather than on its stability, indicating that the mobile active site attracts a substrate in order to reduce its free energy [3]. While flexibility and mobility at the active site enhances functionality, it also is potentially destabilizing to the protein tertiary structure as a whole [3].

To address the challenge associated with the trade-off between protein function and stability, protein engineers have spent the past decade and a half researching ways to manipulate highly stable proteins that are better able to tolerate the mutations necessary to confer new functionality. The concept of “mutational robustness” is one which has particularly interested researchers—a robust protein is better able to tolerate a given point mutation than is a less robust protein [4]. This “robustness” can be evaluated by introducing such a point mutation to the protein and evaluating the ΔΔG_folding_ between the native protein and the mutated protein [4]. ΔΔG_folding_ is defined as the change in ΔG_folding_ between the mutant protein and the parental, or wild-type, protein: ΔΔG_folding_ = ΔG_folding(mutant)_ − ΔG_folding(wild type)_. ΔΔG_folding_ values are direct measures of thermodynamic protein stability: once a mutation has been introduced, the higher (or more positive) the ΔΔG_folding_, the less stable the protein has been rendered as a result of that introduction. Since most function-inducing mutations increase the ΔG_folding_ of a protein, after the introduction of a functional mutation the more robust proteins will have a ΔΔG_folding_ lower than that of less robust proteins [4].

A positive correlation between protein robustness, thermodynamic stability, and “evolvability” has directed research towards utilizing thermophilic proteins as scaffolds for protein engineering [5]. Protein “evolvability” is defined by the ability of a protein to survive mutations which confer new functionality; it has been found that proteins with higher *thermostability* are much more evolvable than are less thermostable proteins. Protein engineers have explored various ways to capitalize upon the correlation between thermostability and evolvability by introducing mutations to thermophilic proteins [5]. The hope is that thermophilic proteins will be an effective and efficient scaffold for functional evolution.

The present article overviews the research that has been performed on thermophilic proteins as potential scaffolds for protein engineering. First, it reviews research which has defined the trade-off between protein activity and stability, and highlights the challenge this trade-off poses to the field of protein engineering. Next, it discusses research describing how protein stability and mutational robustness directly promote evolvability. It then proceeds to introduce thermophilic proteins to this discussion and outline the structural characteristics of these proteins that contribute to their increased stability and mutational robustness. Finally, it highlights recent research which has utilized highly thermostable proteins as effective scaffolds for engineered functional evolution and, when possible, compares the evolvability of these proteins with that of their mesophilic analogues.

## 2. The Trade-Off between Protein Activity and Stability

Over the last decade and a half, numerous studies have demonstrated that there is a distinct trade-off between protein activity and protein stability [2,3]. This trade-off is extremely relevant to the field of protein engineering because it implies that the process of mutating proteins to alter activity can be very destabilizing. In this section, we discuss a comparative analysis of the findings of three important studies which, taken together, demonstrate that mutations that enhance existing protein function may decrease protein stability. The discussion of a fourth study by Tokuriki et al. then demonstrates that those mutations which confer enhanced or new function (“functional” mutations) are more destabilizing than random “neutral” mutations.

Wang et al. studied the evolution of an enzyme, Temoniera-1 (TEM-1) β-lactamase, which is responsible for bacterial antibiotic resistance [6]. In this study, the authors selectively mutated the TEM-1 β-lactamase and observed both the resulting changes in enzymatic activity and in protein stability. Seven different TEM-1 β-lactamase mutants showing increased β-lactamase activity were studied; the authors found that these mutants exhibited a decrease in protein stability relative to the wild-type protein ranging from 0.3 kcal/mol to 4.2 kcal/mol [6]. X-ray crystallographic analysis of the mutant protein structures revealed that the mutations had enlarged the “active site cavity” of the protein—a structural alteration which apparently destabilized the entire three-dimensional structure. Wang et al. also found that the mutants which managed to survive this structural alteration featured secondary mutations which counterbalanced the destabilizing effect of the functional substitutions [6]. This data strongly indicates that a negative correlation between activity and stability may exist.

Bloom et al. computationally evaluated the destabilizing effects of functional mutations on protein structure. Specifically, the authors simulated protein evolution for improved ligand binding affinity by introducing mutations to 20 “lattice” proteins [5]. These lattice proteins were simplified protein models which could be used to simulate protein folding and evolution [5]. The authors found that among all mutated mesophilic proteins only 35% folded to their native three-dimensional structure and that the average ΔG_folding_ for these mutated proteins was higher than that observed for the wild-type protein [5]. The data indicate that proteins which mutate and evolve for new functionality are generally destabilized by the changes which confer the desired functionality. Many of these proteins lose the ability to fold to their native structure entirely, and of those proteins that do retain the ability to fold back to the native structure the average ΔG_folding_ is a less negative value than it had been for the original protein.

Liang et al. examined the tradeoff between protein activity and stability with two related metalloproteinases (MMP): MMP-3 and MMP-12. The authors evaluated the proteinase activity of these enzymes, and found that MMP-12 demonstrated a higher second order rate constant (*k*_cat_/*K*_m_) for the catalytic process than MMP-3 [7]. In contrast, MMP-3 was found to have a catalytic domain 2.8 kcal/mol more stable than that for MMP-12 [7]. The authors evaluated the structures of the two protease domains and noted that MMP-3 displayed a high number of proline residues located at “exposed turns” (or portions of the protein where the primary chain folds back on itself at the exterior of the tertiary structure); this feature accounted for 0.7 kcal/mol of the increased MMP-3 stability [7]. The remaining 2.1 kcal/mol of stability were attributed to the same structural characteristics evaluated by Wang et al.: the increased concentration of buried reactive residues in the vicinity of the active site destabilizing the tertiary network [7]. Liang et al. compared their findings to a previous study performed on MMP-13 (collagenase 3) and MMP-1 (collagenase 1) which similarly noted an increase in activity but a decrease in stability for MMP-13 with respect to MMP-1 [7]. Both of these studies confirm the correlation between increased activity and decreased stability.

While the findings of the previous three studies undoubtedly demonstrate that mutations that enhance function may reduce stability, none of these conclusively show that the “functional” mutations are more destabilizing than random, “neutral” mutations. Tokuriki et al. designed their study to address this [8]. The authors performed their analysis computationally using the folding simulation FoldX to analyze the ΔΔG_folding_ values of 22 mutated proteins with respect to the parental proteins. The proteins that were selected for this study demonstrated the ability to fold to their native structure when either neutral or functional mutations were introduced [8]. Interestingly, analysis of the proteins which underwent functional mutations and subsequently folded to their native state revealed that these proteins also uniformly evolved “other”—or secondary—mutations in addition to the functional mutations [8]. A closer examination of these “other” mutations revealed that they were stabilizing mutations which were largely located on the exterior surface of the protein [8]. It is important to note that the proteins which had undergone neutral mutations had far fewer such stabilizing mutations than did those that were subjected to functional mutations [8]. This discrepancy indicates that the functional mutations, most of which were substituted polar amino acids in the interior of the protein either near or within the active site, were inherently destabilizing to the protein. In order for proteins subjected to these mutations to retain their stability they required additional mutations to counterbalance the destabilizing influence of these substituted residues [8].

## 3. Stability Promotes Evolvability

It is well known that biological processes evolve to function optimally in the surrounding conditions [9]. The evolution of biological process depends greatly upon the evolution of proteins which regulate and control those processes. Thus, for the past two decades researchers have been examining how proteins evolve and how their evolution affects the biological processes which they catalyze and control. By definition, in order for a protein to evolve, it must acquire *new* functionality primarily through the process of mutation. However, as discussed above, this process of protein mutation is likely to be destabilizing. A protein that is stable enough to survive this process and support mutations that bestow new functionality is said to have higher “evolvability” [9]. The following three key papers demonstrate this positive correlation between protein stability and protein evolvability.

The first of these papers, a critical review written by Camps et al., consolidated previous research and identified three protein characteristics that strongly correlate with increased evolvability. The first of these characteristics is protein “promiscuity” which is defined as the ability of a protein “{to recognize} alternative substrates or catalysts or alternative chemical reactions” [9]. Camps et al. explained that a “promiscuous” protein will be more evolvable than other proteins because these proteins require fewer amino acid substitutions to develop new functionality. Clearly a protein that requires six amino acid substitutions to develop new activity will be more likely to evolve functionality than will a protein that requires 40 amino acid substitutions [9]. The second characteristic which Camps et al. correlated with increased evolvability is “modularity” which is defined as “the presence of functionally independent motifs” [9]. Camps et al. reported that a protein featuring many unique, independent subdomains might develop a functionality in one of the domains which is not associated with existing functions, such as enzyme activity [9]. The authors argued that an enzyme subdomain with no existing active site usually needs to undergo fewer destabilizing mutations to develop an active site for a new substrate than an existing active site must undergo to develop new substrate affinity [9].

The third and most important characteristic which the authors correlated with increased protein evolvability is mutational robustness, which is defined as the ability to tolerate mutations [9]. Camps et al. described that there are two subtypes of mutational robustness: “extrinsic” and “intrinsic” [9]. Extrinsic robustness is afforded a protein through “chaperone proteins” or interactions with other nearby proteins [9]. On the other hand, intrinsic robustness is derived from specific characteristics of the protein itself which allow it to more effectively withstand the potentially destabilizing influence of what Tokuriki et al. termed “new function” mutations [8,9]. Intrinsic robustness is thus a direct function of the inherent stability of the protein, so it follows that protein evolvability is directly correlated with protein stability.

This correlation is supported by data from a number of studies which have confirmed that protein mutational robustness and stability increases evolvability. Caetano-Anolles et al. employed an interesting experimental method to evaluate the influence of protein stability on evolvability. The authors inserted a “test protein” into the amino acid sequence of various β-lactamases, which were then expressed in *Escherichia coli*. [10]. It was found that the bacteria expressing more stable β-lactamases (i.e., lower ΔΔG_folding_ values on insertion of the test protein) became resistant to a wider range of penicillin derivatives [10]. Their result is supportive of the view that more stable proteins were better able to accommodate new functional mutations [10].

Another study confirming the existence of the positive correlation between protein stability and evolvability was performed by Philip et al. who experimentally manipulated the functionality of the photoactive yellow protein (PYP)—a 125 residue photoreceptor prototype of the period circadian protein-aryl hydrocarbon receptor nuclear translocator protein-single-minded protein, or PER-ARNT-SIM (PAS) signaling superfamily. PAS proteins are widely known to be extremely diverse—over 20,000 different individual PAS domains have been defined in thousands of signaling proteins in a diverse array of organisms ranging from bacteria to humans [11]. The authors explored the diversity of this class of proteins to determine which structural elements were responsible for its pronounced mutational robustness [11]. They systematically altered each of the 125 residues of this protein one by one, replacing all non-Ala residues with Ala, and all residues with Gly, and developed these mutants in 125 separate *E. coli* strains. Since PYP is a photoreceptor, the authors were able to effectively measure alterations in protein functionality based upon variations in four criteria: visible absorbance maximum, pKa, fluorescence quantum yield, and lifetime of the unstable “pB”—or blue-shifted absorbance—state [11]. These variations were assessed alongside two selected measures of protein stability: variation in ΔG_U_ values (a measure of thermodynamic stability against unfolding) and protein production level [11]. The authors found that many of the substitutions they introduced induced notable alteration in the four selected functional measures, confirming the strong mutational potential of the PYP protein [11]. They also found that alterations specifically at many of 23 specific residues known to be highly conserved throughout PAS domains—most of them far from the active site—either significantly lowered the ΔG_U_ of the PYP protein or decreased overall protein production [11]. From this, they concluded that the strong mutational robustness of the PAS domain derives from the fact that mutations at so many of its residues alters the functionality of the protein, and that a small, key group of highly conserved residues provides it with the stability necessary to support such mutations [11].

## 4. Thermophilic Proteins and Their High Thermostability

Due to the trade-off between protein stability and function, it is highly difficult to introduce mutations necessary for functional evolution without significant compromise of protein stability. Since proteins characterized by higher mutational robustness are more evolvable, as described above, those featuring high mutational robustness and stability could be an important structural framework for functional evolution. One group of proteins which could satisfy the stability requirements is the group of proteins stable at very high temperatures, namely thermophilic and hyperthermophilic proteins. Thermophilic proteins are defined as proteins derived from organisms with optimum growth temperature (OGT) from 45–80 °C, while hyperthermophilic proteins are derived from organisms with OGT above 80 °C (mostly from archaeal lineages) [12]. Mesophilic proteins, on the other hand, are associated with organisms with OGT between 15–45 °C [12]. A number of studies over the past decade have demonstrated that the enhanced stability of thermophilic and hyperthermophilic proteins is characterized by their ability to resist irreversible unfolding when subjected to denaturing conditions. For example, a thermophilic protein unfolded when subjected to heat and chemical denaturants but then refolded into a structure nearly identical to its native state after the denaturing agent was removed [13,14,15]. Many studies have been performed to tease out the factors contributing to enhanced stability of thermophilic proteins. Several comparative studies have recently pinpointed four significant structural differences between thermostable proteins and their mesophilic analogues which go a long way towards explaining the increased thermostability of the thermophilic proteins. These studies demonstrate that the etiology of this enhanced stability is multifactorial: thermophilic proteins feature an increased number of ion pairs, greater average surrounding hydrophobicity of buried side chains, more compact tertiary structure cores, and more hydrogen bonds bridging buried and exposed regions when compared with their mesophilic analogues [16,17,18,19,20,21].

The first major structural difference between thermophilic proteins and their mesophilic analogues is the number of ion pairs [16]. Szilagyi and Zavodszky compared the structures of mesophilic proteins with those of related “moderately thermophilic proteins” (corresponding to the proteins defined earlier in this article as “thermophilic”) and “extremely thermophilic proteins” (corresponding to those defined earlier in this article as “hyperthermophilic”). They found a strong positive correlation between thermostability and the number of ion pairs included in the protein structure [16]. Szilagyi and Zavodszky also found that extremely thermophilic proteins are characterized by stronger ion pairs than their mesophilic counterparts (the strength of an ion pair was defined by the distance between ions: ion pairs separated by less than 4 Å were classified as strong). The authors noticed that many of the ion pairs in extremely thermophilic proteins were separated by distances less than this 4 Å cutoff [16]. By comparison, the separation between most of the ion pairs in moderately mesophilic proteins was on the order of 6 to 8 Å [16]. A follow-up study on the structural characteristics of thermophilic proteins by Gromiha et al. confirmed the findings of Szilagyi and Zavodszky, noting that 68% of thermophilic proteins featured significantly more ion pairs separated by less than 4 Å than did their mesophilic analogues [17].

The second major structural difference between thermophilic proteins and their mesophilic analogues is the average hydrophobicity of the amino acid side chains buried within the protein [17,18]. Gromiha et al. found that a “hydrophobic environment” was the greatest single defining structural element for all of the thermophilic proteins examined, noting that “80% of the thermophilic proteins examined were characterized by higher hydrophobicity than their mesophilic counterparts” [17]. In this study the “surrounding hydrophobicity” of an amino acid side chain was evaluated using a formula, which sums the “hydrophobic indices” of all of the residues within an 8 Å radius of that side chain. This sum was then normalized by the total number of residues in the protein to give the “average hydrophobicity.” Gromiha et al. found that the average hydrophobicity of residues inside the protein is higher in thermophilic proteins (18.5 kcal/mol) than in corresponding mesophilic proteins (17.7 kcal/mol). In contrast, the average hydrophobicity of the exterior residues was similar between thermophilic and mesophilic proteins [17]. That same year, Takano et al. observed that esterase mutants isolated from thermophilic and hyperthermophilic archaea retain their stability at higher temperatures than do similar esterase enzymes derived from mesophilic bacteria [18]. Interestingly, some of the mutants derived from thermostable enzymes demonstrated up to 1.8 times greater relative activity compared to the corresponding wild type enzyme, while no mutants derived from mesophilic enzymes showed any increased activity [18]. The thermophilic enzymes were found to be characterized by buried residues with significantly greater average hydrophobicity than those of the mesophilic counterparts [18]. Unsurprisingly, given this finding, the mutants which were derived from thermophilic enzymes but found to be unstable were disproportionately those which underwent substitution of interior residues: 90% of the destabilized thermophilic mutant proteins in this study had interior residue substitution; only 30% of the thermophilic mutant proteins which retained stability had interior residue substitution [18]. Taken together, both of these studies clearly demonstrate the role of average hydrophobicity of interior residue side chains in the stabilization of thermophilic proteins. One potential explanation for these findings is that the hydrophobic residues packed into the core of the protein tightly adhere to one another and resist the destabilizing influence of the external environment.

The third major structural difference between thermophilic proteins and their mesophilic analogues is the presence of compact, stable structural cores [19,20,21]. Meruelo et al. found that, when compared to mesophilic analogues, thermophilic proteins consist of a higher quantity of small amino acids such as Gly, Ala, Ser, and Val and a smaller quantity of large and/or polar amino acids such as Cys, Asp, Glu, Gln, and Arg [19]. The authors note that the smaller number of bulky, reactive amino acid side chains allows for tighter packing as the protein folds into its tertiary structure, and greater resistance to unfolding and aggregation. This corresponds to the findings of Glyakina et al. where interior residues of thermophilic proteins were more tightly packed, when compared to their mesophilic counterparts [20]. Tompa et al. expanded on this work by demonstrating that even when residues with long, hydrophobic side chains are heavily represented in thermophilic proteins, these residues are packaged in the core of the tertiary structure in such a way where residue-residue contact is maximized, thereby tying the core into a tight, compact, and stable structure [21]. The importance of a compact tertiary core to protein stability is illustrated further by research which demonstrates that hyperthermophilic proteins have significantly more disulfide bonds than either mesophilic or thermophilic proteins [19,22,23]. This feature serves to maximize the tight binding arrangement of core protein residues and optimize tertiary stability. Taken together, the research cited above support the notion that tight residue packing promotes greater interactions between neighboring residues and thereby enhances thermostability.

The fourth major structural difference between thermophilic proteins and their mesophilic counterparts is the number of hydrogen bonds bridging a protein’s buried and exposed regions. The study performed by Tompa et al. showed that the distribution of hydrogen bonds is different in thermophilic proteins compared to that seen in their mesophilic counterparts [21]. The researchers noted that 49% of the hydrogen bonds found in thermophilic proteins bridged residues that were buried within the core of the protein with residues located on the exterior of the protein—only 42% of hydrogen bonds in mesophilic proteins met those criteria [21]. Additionally, 49% of hydrogen bonds in thermophilic proteins were found to exist between the protein main chain (the amino acid backbone chain) and side chain residues; this compares with only 39% in mesophilic proteins [21]. Hydrogen bonds are a particularly strong and stable form of intermolecular force—the authors theorize that each of the described altered hydrogen bond distributions in thermophilic protein conveys enhanced stability in its own way. First, the higher percentage of hydrogen bonds between buried and exposed residues effectively tethers the more vulnerable and thus unstable exterior of the protein to its compact, protected core. Second, the higher percentage of hydrogen bonds between the backbone amino acid chains and the side chains effectively crosslinks all elements of the tertiary structure in a fashion less vulnerable to denaturation [21].

**Figure 1:**
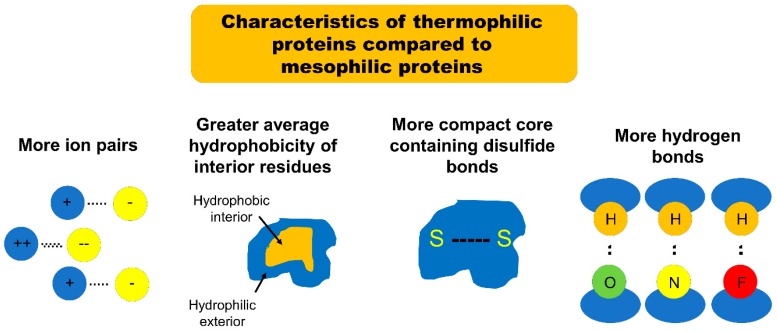
A schematic of characteristics of thermophilic proteins when compared to mesophilic proteins.

In summary, the increased thermostability of thermophilic proteins appears to be attributable to an increased number of strong ion pairs, an increased surrounding hydrophobicity of buried amino acid side chains, more compact cores, and a more consolidating distribution of hydrogen bonds with respect to similar mesophilic proteins (Figure 1). It might initially appear that an increased number of ion pairs is contradictory to an increased average hydrophobicity. However, the two characteristics are compatible; the non-ionic residues in thermophilic proteins have a significantly greater surrounding hydrophobicity than do those in their mesophilic counterparts [16].

All of the above features contribute to the stabilization of thermophilic and hyperthermophilic proteins by increasing the rigidity of the corresponding tertiary structures. Rigidity and stability are correlated when it comes to protein structures; thermophilic and hyperthermophilic proteins can afford to be rigid, because they operate by definition in high temperature environments which develop sufficient interactions between substrates and active sites with limited flexibility [24]. These proteins are tightly packed, inflexible, and unfold much more slowly than do their less thermostable counterparts [25,26]. This discussion warrants mentioning psychrophilic proteins which stand on the other end of the rigidity/stability spectrum. These proteins have flexible tertiary structures which facilitate necessary interactions with substrates even at low temperatures [24,27]. Protein flexibility is a positive attribute under certain circumstances: it is vital for protein function in low temperature environments and it may permit singular proteins to interact with a wider range of substrates than can more rigid proteins. However, the proteins of most use to protein engineers are those able to withstand dramatic manipulation and retain functionality. Increased rigidity, and thus stability, make thermophilic and hyperthermophilic proteins more evolvable and subsequently invaluable in this capacity.

It is worth noting at this point that the magnitude of contribution from denaturation entropy changes to the enhanced stability of thermophilic and hyperthermophilic proteins is a topic of debate. This is an empirically observed phenomenon which specifies that the ΔS values associated with the denaturation of thermostable proteins are smaller than those associated with denaturation of mesophilic proteins [28]. The hypothesized rationale for this is that thermostable proteins have a higher baseline entropy in their native state compared to mesophilic proteins [28]. Wintrode et al. showed that thermophilic proteins have increased populations of low-frequency vibrational states largely due the high incidence of ion pairs and charge-charge networks on the surface of these proteins, and that this contributed to the baseline entropy level of the native state of thermophilic proteins [29]. Berezovsky et al. showed that thermophilic proteins are characterized by high numbers of lysine residues buried inside the protein core [30]. This is significant because buried lysine residues have a much higher density of variable rotamers than do other charged residues (such as arginine): this also contributes to the overall baseline entropy of the native state of thermophilic proteins [30]. These findings point to a potentially important entropic contribution to thermophilic protein stability. How much of overall thermophilic protein stability is attributable to enhanced structural rigidity (as discussed above) and how much is attributable to this entropic phenomenon is still under discussion. Regardless, the increased stability of thermophilic proteins provides some uniquely exciting opportunities in the protein engineering field. Most importantly, a protein characterized by increased thermostability may also feature a high level of mutational robustness and is thus better able to accommodate functional evolution than is a protein with lower thermostability.

## 5. Thermophilic Proteins as a Scaffold for Functional Evolution

Since thermophilic proteins generally exhibit a higher level of mutational robustness when compared with their mesophilic counterparts, it stands to reason that these proteins are natural candidates to become scaffolds for evolutionary engineering. A number of studies have therefore been performed over the past decade evaluating thermophilic proteins as scaffolds for protein engineering and exploring options for application. This literature has become more robust recently as studies have begun to directly compare the evolvability of thermophilic proteins with that of analogous mesophilic proteins.

Bloom et al. randomly mutated two separate variants of a cytochrome P450 BM3 heme domain peroxygenase and screened the resulting mutants for new functionality (in this instance, the hydroxylation of various antibiotic drugs) [5]. The two variants examined were the mesophilic 21B3 enzyme (the temperature at which the protein loses 50% activity (*T*_50_) = 47 °C) and the thermophilic 5H6 enzyme (*T*_50_ = 62 °C) [5]. The authors found that the increased thermostability of the 5H6 enzyme allowed more mutants to fold (61% of the total) than did the 21B3 parent (34%) [5]. This data corroborates previous evidence that increased thermostability is correlated with increased ability to tolerate mutations. The authors also observed that the 5H6 mutants developed new functionality at a higher rate than did the 21B3 mutants. Citing this data, Bloom et al. argued that the increased mutational robustness of thermophilic proteins makes them more effective scaffolds for protein engineering than their mesophilic analogues [5]. A study performed by Bershtein et al. confirms the correlation asserted by Bloom et al. between protein thermostability and evolvability by mutating a thermophilic variant of TEM-1 β-lactamase to develop new functionality [31]. The authors used error-prone polymerase chain reaction (PCR) to induce “neutral drift” (the generation of active, mutant variants which retain the same function as the original protein) among the protein population [31]. The mutant variants which were characterized by higher *T*_50_ values were isolated from the rest and subsequently tested for new functionality. Interestingly, the isolated variants were found to have evolved new functionality against cephalosporin antibiotics: these thermophilic variants were roughly 800 times more efficient at degrading the cephalosporin cefotaxime than were the original parent TEM-1 β-lactamase [31]. Since the authors took great care to ensure a neutral drift among the TEM-1 β-lactamase variants, the parent proteins serve as mesophilic analogues to the thermophilic variants in this paradigm. Overall, their finding that the thermophilic variants exhibited new functionality which was lacking both in the parent protein and in mesophilic analogues demonstrates the benefit of high thermostability for facile functional evolution [31].

Another experimental example of thermophilic proteins being utilized as scaffolds for protein evolution was reported by Tokuriki and Tawfik [32]. In this study, the authors subjected GADPH and CAII to error-prone PCR in the presence and absence of GroEL/GroES heat-shock chaperonins isolated from *E. coli* bacteria [32]. Previous literature had established the selected chaperonins as vitally important in increasing the ability of proteins to survive at high temperatures [32]. The protein variants developed in the presence of the chaperonins were then compared with those developed in the absence of chaperonins. As anticipated, the protein’s mutational robustness was enhanced upon addition of chaperonins; the variants evolved with chaperonins were able to accommodate the incorporation of destabilizing mutations with ΔΔG_folding_ of up to 3.5 kcal/mol, whereas those developed without chaperonins were only able to tolerate destabilizing mutations with a ΔΔG_folding_ of up to 1 kcal/mol [32]. In addition, the variants developed with chaperonins were identified as “adaptive functional” variants—that is, mutants which retained the ability to fold but demonstrated new function—at a higher frequency than those developed without chaperonins [32].

Subsequent research performed by Aledo et al. compared the thermostability of two mammalian protein analogues: cytochrome b and cyclooxygenase I (COX I) to examine a correlation between evolvability and thermostability. Whereas previous studies done to evaluate the relationship between thermostability and evolvability started with a known thermophilic protein and a known mesophilic protein and compared their evolvability, this study does the opposite. The authors started with a pair of proteins, cytochrome b and COX I, known respectively to be highly evolvable and poorly evolvable, and compared their thermostability [33]. Both proteins were virtually mutated and aligned on the ClustaIX bioinformatics platform and the destabilizing effect of each mutation on the protein were evaluated [33]. Aledo et al. found that the average destabilizing effect of the mutations on the cytochrome b protein was ~1 kcal/mol, while that on the COX I protein ranged from 1.5–1.9 kcal/mol [33]. The average destabilizing effect of mutations on cytochrome b fell within a range of the degree by which mutations destabilize known thermophilic proteins. Likewise, the average destabilizing effect of mutations on COX I was quantitatively similar to the effect of mutations on known mesophilic proteins. Thus, the more evolvable protein exhibits similar mutational robustness to known thermophilic proteins whereas the less evolvable protein exhibits similar mutational robustness to known mesophilic proteins; these findings supports the assertion that thermophilic proteins are better scaffolds for protein engineering than are mesophilic proteins.

Takahashi et al. identified a thermophilic variant of D-amino acid oxidase (DAO) in the thermophilic bacterium *Rubrobacter xylanophilus* and explored its applicability as a stable substitute for the ubiquitous DAO variant found in eukaryotes [34]. DAO is a biotechnologically attractive enzyme utilized for a variety of applications in biomedical science; it is, however, extremely unstable. This fact circumscribes its applicability and limits its potential for introduced functionality. Importantly, the thermophilic variant of DAO remained active not only at an elevated temperature but also at a low pH unlike the eukaryotic, mesophilic DAO, and also retained enzyme activity in the presence of certain thiol-modifying reagents known to inhibit the mesophilic variant [34]. The authors assert that these findings render the thermophilic DAO a strong candidate for functional engineering which would expand the applicability of the DAO family beyond the current capabilities of the mesophilic DAO [34].

Thermophilic proteins have also proven themselves superior scaffolds for the branch of protein engineering that explores protein fragmentation and cooperative function. This is a particularly challenging area of protein engineering which involves the destruction of parent proteins in such a manner that the resulting fragments are capable of reassembling themselves into stable, functional tertiary structures similar to the parents [35]. It is unsurprising that hyperstable thermophilic and hyperthermophilic proteins have shown themselves to be superior scaffolds for such potentially destabilizing interruptions. Nguyen et al. first showed that split adenylate kinases from the hyperthermophilic *Thermotoga neapolitana* were able to form functional complements which efficiently supported the growth of *E. coli* in culture; similar fragments from the mesophilic *Bacillus subtilis* were not able to do the same [36]. The authors also found that the degree of enzyme fragment complementation and function was directly proportional to the midpoint for thermal denaturation (i.e., melting temperature (*T*_m_)—a measure of protein themostability) of the parent enzyme: a consequence of the fact that the enzyme fragments derived from the hyperthermophilic parents retained more of the original tertiary structure of their parents than did their mesophilic fragment counterparts [36]. A follow-up study performed by Segall-Shapiro et al. on the same enzymes characterized this dichotomy further by finding that 44% of hyperthermophilic fragments generated in their study were capable of forming composites that supported *E. coli* growth, whereas only 6% of mesophilic fragments were capable of doing the same [37]. The authors attributed this finding to the fact that truncation of the hyperthermophilic protein yielded both more functional fragments and more unique fragment variants overall (41% of total fragments) than did truncation of the mesophilic parent (30% of fragments) [37].

More recently, a series of studies performed by Kim and coworkers demonstrated that domain insertion of a guest protein may benefit from high thermostability of a host protein [38,39,40,41]. Insertional fusion has recently been highlighted as a novel means of creating multi-domain protein complexes, where functionalities are often integrated and coupled with each other. In this type of fusion, a guest protein domain is inserted into the middle of a host protein domain. Unfortunately, due to the disruption of a host protein’s primary sequence upon domain insertion, the insertional fusion is energetically challenging and often leads to significant compromise of protein stability [39]. To overcome such energetic penalty, Kim and coworkers used a thermophilic maltodextrin-binding protein from *Pyrococcus furiosus* as a host protein, into which various guest enzyme domains, such as exoinulinase, TEM-1 β-lactamase and xylanase, were successfully inserted. The insertional fusions led to the creation of chimeric protein complexes, where thermostability of guest enzyme domains was improved by various mechanisms [38,39,40,41,42]. In contrast, similar insertional fusion into a mesophilic maltodextrin-binding protein from *E. coli* lowered thermostability and expression levels of a guest enzyme domain [39]. In addition, functional evolution for enhanced enzyme activity toward alkaline pH of *Bacillus circulans* xylanase was greatly assisted by its enhanced thermostability acquired by the insertional fusion [42]. The implication is that the thermophilic host protein could serve as a stabilizing scaffold allowing the introduction of many gain-of-function mutations to multiple guest proteins, which would otherwise be catastrophically destabilizing [42].

The research discussed above strongly supports the notion that high stability of thermophilic proteins makes them greatly suitable for protein engineering, when compared to their mesophilic analogues. The applications for these highly stable proteins are clearly manifold in the field of protein engineering as they provide a broad array of exciting opportunities for the protein engineers of the new century.

## 6. Conclusions

The field of protein engineering is one which endeavors to introduce new functionality to protein domains. Since there is an inherent trade-off between protein functionality and protein stability, protein engineers must constantly balance the destabilizing effect of the mutations they introduce to protein domains with the anticipated modifications they are attempting to make to the protein. Over the course of the past decade and a half, many studies have shown that one of the best ways to increase a protein’s chance of accommodating a functional mutation is to have an increased stability or mutational robustness to begin with. Numerous studies first confirmed that proteins characterized by greater stability were better able to withstand mutations, and later demonstrated that they were better able to support the type of mutations required to enhance existing function or introduce new function. The positive correlation between protein stability and evolvability was thus established.

Thermophilic proteins are characterized by certain significant structural differences from mesophilic proteins and often are accompanied by “chaperone proteins” which help the protein maintain its three-dimensional structure at high temperatures. Over the past 20 years, a nascent but increasingly robust literature has been developed directly comparing the evolvability of thermophilic proteins to that of mesophilic proteins; this literature clearly demonstrates that thermophilic proteins are indeed more evolvable and more tolerant of mutations introduced to enhance or modify functionality. This renders the thermophilic proteins superior scaffolds for protein engineering.

**Figure 2:**
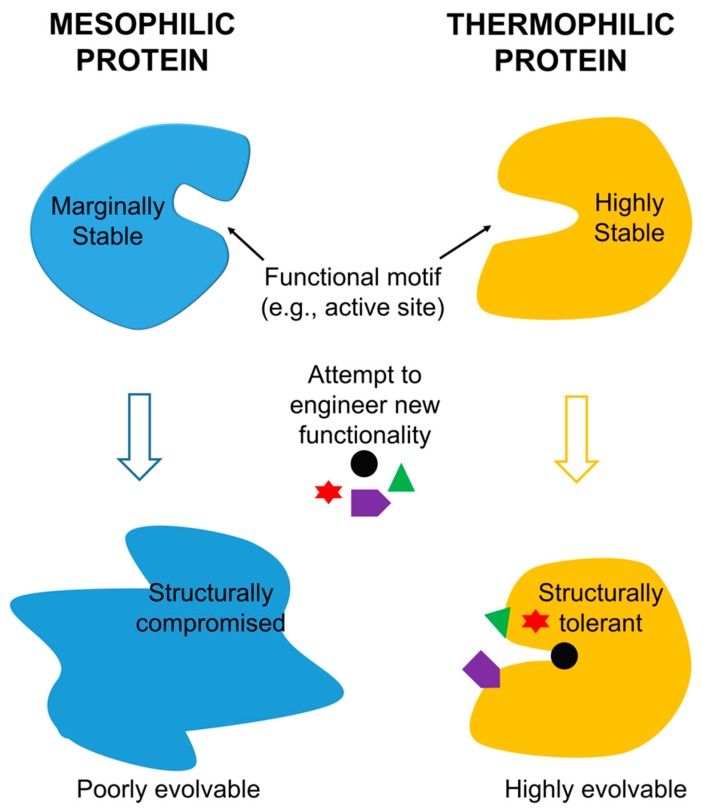
A schematic of facile functional evolution from thermophilic proteins assisted by their high thermostability. Insufficient stability of mesophilic proteins limits their evolvability.

Thermophilic proteins thus appear to be an efficient and effective solution to the challenge posed by the simultaneous manipulation of protein function and maintenance of protein stability. Functionality and stability do indeed trade off, stability does indeed promote evolvability, and the increased stability of thermophilic proteins does indeed make this kind of protein a uniquely effective scaffold for evolving protein function, as summarized in Figure 2. Thermophilic proteins will likely be the scaffold of choice for the protein engineers of the future.
